# A predictive model for early neurological deterioration after intravenous thrombolysis in patients with ischemic stroke

**DOI:** 10.3389/fneur.2025.1477286

**Published:** 2025-02-17

**Authors:** Liping He, Meng Zhang, Fei Xu, Zhangsong Wu, Huijuan Chen, Ying Li, Ran Chen

**Affiliations:** ^1^Department of School, Bengbu Medical University Graduate School, Bengbu, China; ^2^Department of Neurology, Anhui Medical University Lu’an People’s Hospital, Lu’an, China

**Keywords:** acute ischemic stroke, intravenous thrombolysis, machine learning, early neurological deterioration, predictive models, nomogram

## Abstract

**Objective:**

Intravenous thrombolysis (IVT) is the treatment of choice for acute ischemic stroke (AIS), but some patients develop early neurological deterioration (END) within 24 h after IVT. Therefore, we aimed to identify predictors of END in AIS patients following treatment with IVT.

**Methods:**

We retrospectively analyzed the clinical data of 621 AIS patients who received IVT with recombinant tissue-type plasminogen activator (rt-PA) at the Stroke Centre of the People’s Hospital of Lu’an City, China, from July 2018 to July 2023. Clinical data, including demographic characteristics, clinical assessment results, underlying diseases, and laboratory indices, were collected at the time of admission. The patients were divided into training and validation cohorts, after which LASSO regression was applied to select the most important predictor variables, and multivariate logistic regression was used to construct a nomogram. The discriminative power of the model was determined by calculating the area under the curve (AUC), and calibration and decision curve analyses (DCA) were performed.

**Results:**

The platelet-to-lymphocyte ratio (PLR) (OR 1.01, 95% CI 1.01–1.01, *p* < 0.001), mean platelet corpuscular volume (MPV) (OR 2.12, 95% CI 1.67–2.69, *p* < 0.001), and admission NIHSS score (OR 1.25, 95% CI 1.16–1.36, *p* < 0.001) were significantly associated with the development of END. The AUC of the prediction model constructed from these three factors was 0.896 (95% CI 0.862–0.93), and the calibration curve was close to the diagonal.

**Conclusion:**

This predictive model can be used for the early identification of the risk of developing END after IVT and development of active interventions to improve the prognosis of AIS.

## 1 Introduction

Ischemic stroke is the most common type of stroke in China and is a major cause of death in patients. Statistics show that ischemic stroke accounts for 60 to 80 percent of stroke cases in China. To improve the therapeutic outcomes of ischemic stroke patients, acute reperfusion therapy is considered to be the current method of choice (1). However, despite the clinical success of this treatment modality, approximately 10 to 15 percent of patients undergoing intravenous thrombolysis (IVT) experience early neurological deterioration (END). END, defined as further deterioration of the disease and additional neurological damage within 24 h, has a serious impact on patients’ neurological function and often has a poor prognosis (2). The literature suggests that a variety of factors such as high blood glucose levels, higher baseline National Institutes of Health Stroke Scale (NIHSS) scores, and higher systolic blood pressure values may be potential predictors of the development of END. However, the predictive value of these indicators has not been fully established (3). Therefore, how to identify and intervene early regarding factors associated with END is important for the treatment and prognosis of ischemic stroke. In order to gain a deeper understanding of these influencing factors and to provide theoretical and practical support for clinical practice, the aim of this study was to analyze the factors associated with END in acute ischemic stroke patients after intravenous thrombolytic therapy. The study assessed the risk of END in patients by collecting rich data on clinical characteristics and constructing a predictive model. Through this study, we hope to be able to provide clinicians with more targeted recommendations for prevention and treatment strategies that will significantly improve the patient outcomes and long-term prognosis of ischemic stroke. This will not only help to increase the survival rate of patients with acute ischemic stroke but also improve their quality of life and reduce the reoccurrence rate and healthcare burden.

## 2 Materials and methods

### 2.1 Study population

In this retrospective study, consecutive patients who were diagnosed with acute ischemic stroke (AIS) and treated with IVT from July 2018 to July 2023 at Anhui Medical University Lu’an People’s Hospital were included. The study was approved by the Ethics Committee of Anhui Medical University Lu’an People’s Hospital and was conducted in accordance with the 1964 Declaration of Helsinki and its subsequent amendments. All patients’ families signed informed consent forms for administering recombinant tissue-type plasminogen activator (rt-PA) IVT.

The inclusion criteria were as follows: (1) 18 years of age or older; (2) use of alteplase thrombolytic therapy and a symptom onset time (defined as the time from symptom onset to thrombolytic therapy) ≤4.5 h; (3) a clear diagnosis of AIS (confirmed by computed tomography or magnetic resonance imaging) and the presence of certain neurological deficits; and (4) agreement that the patient or one of his or her family members would sign an informed consent form.

The exclusion criteria were as follows: (1) hemorrhagic cerebral infarction; (2) transient ischemic attack; (3) bridging endovascular therapy after IVT; (4) brain tumors; (5) lack of complete clinical and laboratory data; and (6) contraindications to IVT (e.g., intracranial hemorrhage, history of intracranial hemorrhage, intracranial tumors, giant intracranial aneurysms, active visceral hemorrhage, platelets <100 × 10^9^/L, oral anticoagulants and international normalized ratio (INR) >1.7 or prothrombin time (PT) >15 s and intracranial or intralesional surgery within 3 months prior to IVT).

### 2.2 Data collection

The following data were retrospectively collected for all patients: age, sex, height, weight, vascular risk factors [hypertension, diabetes mellitus (DM), atrial fibrillation (AF), hyperhomocysteinemia], history of smoking and alcohol consumption, and laboratory test results [i.e., prethrombolytic blood glucose (FBG), neutrophil count, lymphocyte count, erythrocyte count, platelet count, mean platelet volume (MPV), triglycerides (TG), total cholesterol (TC), low-density lipoprotein cholesterol (LDL-C), high-density lipoprotein cholesterol (HDL-C), sodium, potassium, neutrophil-to-lymphocyte ratio (NLR), and platelet-to-lymphocyte ratio (PLR)]. Venous blood samples were collected before IVT on admission. The body mass index (BMI) was calculated as the weight in kilograms divided by the square of the height in meters. NIHSS scores on admission and 24 h after IVT were assessed by two experienced neurologists.

#### 2.2.1 Grouping

Patients were divided into the END group {Group 0} and the non-END group {Group 1} according to the presence or absence of END after IVT. END was defined as an increase of ≥4 points in the NIHSS score within 24 h of admission.

### 2.3 Model construction and verification

The patients included in this study were divided into a training cohort and a validation cohort at a ratio of 7:3 via nonrepeated random sampling. The data from the training cohort were used to construct the model, and data from the validation cohort were used to test the constructed model. None of the variables were significantly different between the two cohorts, indicating that the data were grouped randomly and reasonably. Least absolute selection and shrinkage operator (LASSO) regression, an effective high-dimensional method for improving model predictions, was used to identify the most important predictors among the parameters; when performing LASSO regression, the optimal value of *λ* was determined by tenfold cross-validation. Then, multivariate logistic regression analysis was performed to identify statistically significant predictor variables among those selected in the LASSO regression model. The predictive accuracy and discriminative ability of the resulting nomogram were evaluated using receiver operating characteristic (ROC) curve and calibration curve analyses in the training and validation cohorts, and the clinical applicability of the nomogram was evaluated using decision curve analysis (DCA) (4, 5).

### 2.4 Statistical analysis

Statistical analyses were performed using SPSS 26.0 software. Normally distributed measurement data are expressed as the mean ± standard deviation (x ± s), and comparisons between groups were made using the independent samples *t*-test; nonnormally distributed measurement data are expressed as the median (lower and upper quartiles) [M(QL, QU)], categorical data are described as frequencies (%), and comparisons between groups were made using the *χ*^2^ test or Fisher’s exact probability method. A bilateral *p*-value <0.05 was considered statistically significant. R version 4.3.0 was used to complete the LASSO regression model and construct the nomogram, as well as to plot the ROC curves, calibration curves, and decision curves.

## 3 Results

### 3.1 Patient information

**Figure 1:**
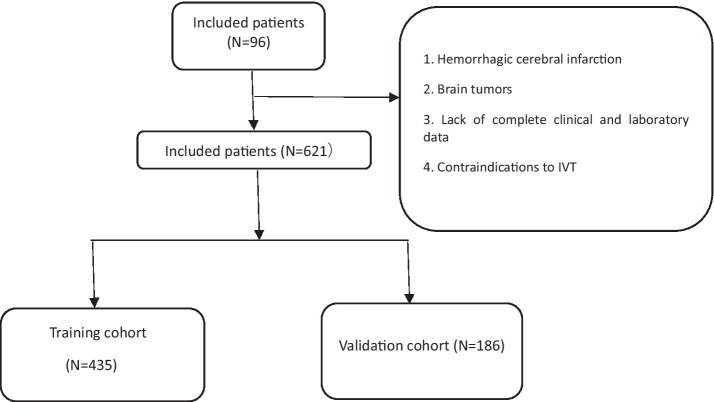
Case selection process.

We enrolled 960 stroke patients who received IVT at the Stroke Centre of Lu’an People’s Hospital between July 2018 and July 2023; patients were excluded due to the presence of a hemorrhagic cerebral infarction, transient ischemic attack, bridging endovascular therapy after IVT, brain tumors, the lack of complete clinical and laboratory examination data, and contraindications to IVT. Finally, a total of 621 patients were included in this study. The baseline data of the 527 patients who did not show early neurological deterioration and the 94 patients who did were compared (Table 1) There were differences in the lymphocyte count, neutrophil-to-lymphocyte ratio, PLR, MPV, and admission NIHSS score. We used nonrepeated random sampling (at a 7:3 ratio) to divide the patients into a training cohort (435 patients) and a validation cohort (186 patients) (Figure 1).

**Table 1:** Comparison of baseline data between AIS patients with and without END after IVT.

Variable	Group 0 (*n* = 527)	Group 1 (*n* = 94)	Statistical value	*p*-value
Age	67.00 (56.00–75.00)	68.00 (59.25–75.75)	1.646	0.2
Height	168.00 (160.00–172.00)	166.00 (158.00–171.00)	0.517	0.472
Weight	66.00 (59.00–74.00)	66.00 (58.00–72.00)	0.585	0.445
BMI	23.73 (22.03–25.79)	23.58 (21.82–26.04)	0.211	0.646
Red blood cell	4.65 (4.29–4.99)	4.65 (4.25–4.96)	0.152	0.697
Neutrophil count	4.76 (3.64–6.11)	4.81 (3.95–6.15)	0.422	0.516
Lymphocyte count	1.67 (1.24–2.24)	1.33 (0.80–1.90)	19.128	< 0.01
White biood cell	7.28 (6.07–8.77)	7.04 (6.00–8.57)	1.244	0.265
Neutrophil-to-lymphocyte ratio	2.72 (1.79–4.48)	3.88 (2.38–5.94)	13.536	< 0.01
Platelet-to-lymphocyte ratio	122.33 (90.42–161.80)	161.08 (110.64–233.57)	24.287	< 0.01
Lymphocyte percentage	23.30 (15.40–31.05)	20.30 (14.90–28.67)	1.726	0.189
Average platelet volume	9.20 (8.50–10.10)	10.60 (10.10–11.58)	95.721	< 0.01
Triglyceride	1.13 (0.80–1.72)	1.21 (0.88–1.90)	2.258	0.133
Total cholesterol	4.91 (4.08–5.55)	5.00 (4.23–5.72)	0.833	0.361
Low-density lipoprotein	2.82 (2.31–3.42)	2.96 (2.27–3.75)	2.229	0.135
Na	139.70 (137.90–141.60)	138.85 (137.50–141.30)	2.49	0.115
K	3.85 (3.53–4.12)	3.82 (3.55–4.09)	0.187	0.666
NIHSS score	3.00 (2.00–5.00)	6.00 (4.25–8.00)	83.361	< 0.01
Blood glucose	6.89 (5.88–8.65)	7.41 (6.43–9.95)	6.057	0.014
Platelets	205.00 (169.00–244.00)	204.00 (170.50–245.50)	0.006	0.94
Sex, *n* (%)			3.348	0.067
0	173 (32.83)	40 (42.55)		
1	354 (67.17)	54 (57.45)		
Hypertension, *n* (%)			0.85	0.356
0	81 (15.37)	11 (11.7)		
1	446 (84.63)	83 (88.3)		
Diabetes, *n* (%)			0.919	0.338
0	421 (79.89)	71 (75.53)		
1	106 (20.11)	23 (24.47)		
Atrial fibrillation, *n* (%)			1.168	0.28
0	436 (82.73)	82 (87.23)		
1	91 (17.27)	12 (12.77)		
Hyperhomocysteinemia, *n* (%)			7.49	0.006
0	314 (59.58)	70 (74.47)		
1	213 (40.42)	24 (25.53)		
Smoking, *n* (%)			0.695	0.404
0	354 (67.17)	59 (62.77)		
1	173 (32.83)	35 (37.23)		
Alcohol consumption, *n* (%)			1.464	0.226
0	348 (66.03)	56 (59.57)		
1	179 (33.97)	38 (40.43)		
Hyperlipidemia, *n* (%)			1.399	0.237
0	395 (74.95)	65 (69.15)		
1	132 (25.05)	29 (30.85)		

### 3.2 Construction of the predictive model based on LASSO Cox regression

**Figure 2:**
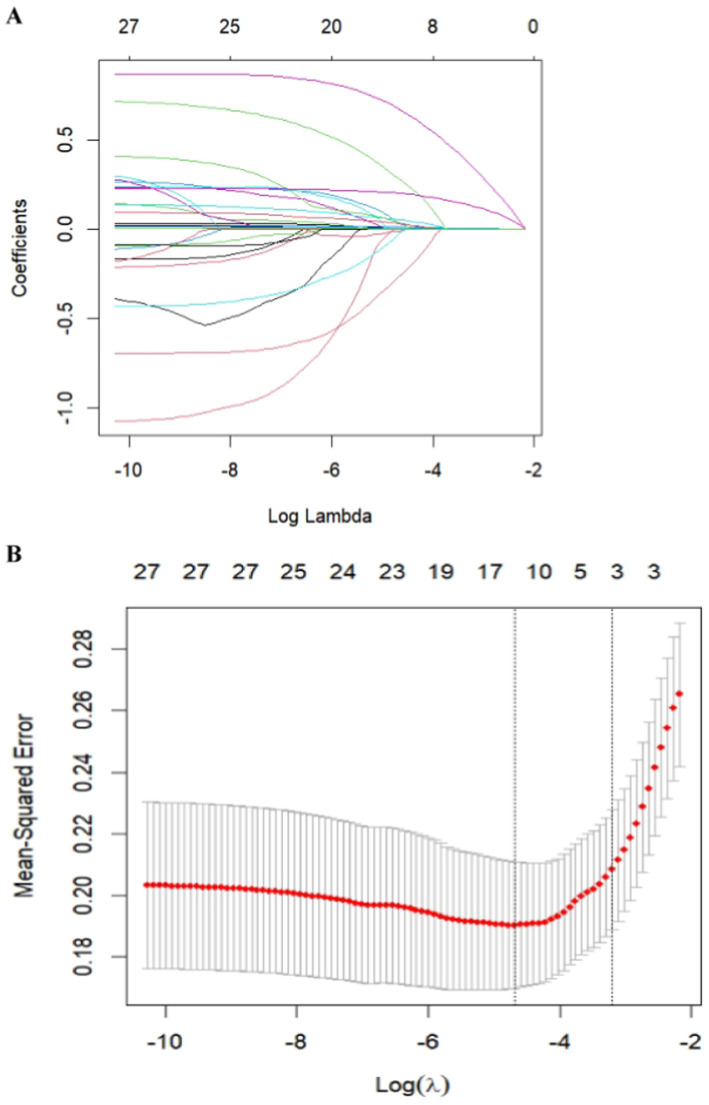
Feature selection was performed using a LASSO binary logistic regression model. **(A)** Log(lambda) values of the 28 features initially included in the LASSO model. Coefficient distribution plots were generated from the log(lambda) series. **(B)** Parameter selection in the LASSO model through the minimum criterion method using tenfold cross-validation. Partial likelihood deviation (binomial deviation) curves and log(lambda) curves are plotted. Vertical dashed lines are drawn at the lambda values using the minimum criterion and 1-se of the minimum criterion (1-SE criterion). The optimal lambda value yielded three variables with nonzero coefficients.

**Figure 3:**
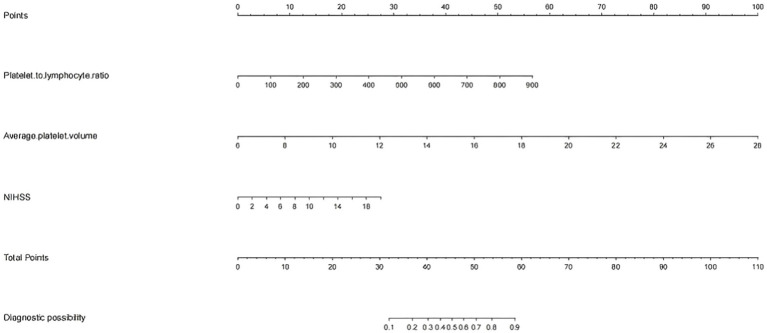
Nomogram for predicting the risk of early-onset neurological deterioration in AIS patients treated with intravenous thrombolysis. For all patients, the points determined on the score sheet for all three indicators were summed. The sum was then placed on the “total score” axis. Finally, the risk of END based on the nomogram is the probability of “END” corresponding to the “total points.” NIHSS, National Institutes of Health Stroke Scale; MPV, mean platelet corpuscular volume; PLR, platelet-to-lymphocyte ratio.

The patients were randomly divided into a training cohort and a validation cohort at a ratio of 7:3. The data from the training cohort were used to build the model, and those from the validation cohort were used to test the constructed model. None of the 28 variables collected from the patients were significantly different (*p* > 0.05), indicating that the data grouping was random and reasonable (Table 2). LASSO regression was used to initially screen the parameters; the changes in the coefficients of these variables with different values of *λ* are shown in Figure 2A. Iterative analysis using 10-fold cross-validation revealed that when the *λ*.1se value was 0.04072893, a model with optimal performance but the least number of variables could be constructed (Figure 2B). *λ*.1se is the value of *λ* that yields the simplest model within one variance of *λ*.min. Because continuing to increase the number of independent variables in the model, i.e., shrinking the *λ*-value, does not significantly improve model performance after a certain *λ*-value is reached, *λ*.1se provides a model with excellent performance and includes the smallest number of independent variables. The selected variables included the PLR, MPV, and NIHSS score at admission (Table 3). The variance inflation factors of the above three variables were 1.137395, 1.159108, and 1.043182, respectively; these values were all less than 10, suggesting the absence of collinearity and thus that these variables could all be included in the multiple logistic regression analysis. The above independent predictors were then employed to develop a predictive nomogram (Figure 3).

**Table 2:** Comparison of baseline information between the training and validation cohorts.

Characteristic	Validation cohort (*n* = 186)	Training cohort (*n* = 435)	Statistical value	*p*-value
Age	67.00 (57.00–74.00)	68.00 (57.00–75.00)	0.292	0.589
Height	165.00 (159.25–171.00)	168.00 (160.00–172.00)	2.95	0.086
Weight	65.00 (57.00–70.75)	66.00 (59.00–75.00)	2.925	0.087
BMI	23.66 (21.93–25.19)	23.82 (22.03–25.95)	1.205	0.272
Red blood cell	4.59 (4.29–4.98)	4.65 (4.29–4.98)	0.223	0.637
Neutrophil count	4.89 (3.80–6.18)	4.76 (3.63–6.11)	0.377	0.539
Lymphocyte count	1.50 (1.10–2.19)	1.64 (1.22–2.21)	3.235	0.072
White blood cell	7.23 (6.07–8.63)	7.27 (6.04–8.82)	0.018	0.894
Neutrophil-to-lymphocyte ratio	3.18 (1.97–4.74)	2.77 (1.80–4.73)	2.69	0.101
Platelet-to-lymphocyte ratio	131.10 (94.22–175.47)	124.76 (91.84–168.56)	1.694	0.193
Lymphocyte percentage	21.65 (15.00–29.82)	23.40 (15.40–31.00)	2.583	0.108
Average platelet volume	9.50 (8.70–10.50)	9.40 (8.60–10.30)	1.79	0.181
Triglycerides	1.08 (0.78–1.62)	1.17 (0.82–1.76)	1.666	0.197
Total cholesterol	4.71 (4.05–5.44)	4.98 (4.13–5.65)	3.505	0.061
Low-density lipoprotein	2.76 (2.29–3.37)	2.86 (2.32–3.54)	2.067	0.151
Na	139.85 (138.20–141.60)	139.40 (137.70–141.60)	2.498	0.114
K	3.82 (3.53–4.09)	3.85 (3.54–4.12)	0.047	0.828
NIHSS score	3.00 (2.00–5.00)	3.00 (2.00–6.00)	0.91	0.34
Blood glucose	6.84 (5.88–8.96)	7.08 (5.94–8.72)	0.008	0.929
Plateles	205.00 (164.50–244.00)	204.00 (170.00–244.00)	< 0.001	0.998
Sex, *n* (%)			0.922	0.337
0	69 (37.1)	144 (33.1)		
1	117 (62.9)	291 (66.9)		
Hypertension, *n* (%)			3.37	0.066
0	35 (18.82)	57 (13.1)		
1	151 (81.18)	378 (86.9)		
Diabetes, *n* (%)			0.887	0.346
0	143 (76.88)	349 (80.23)		
1	43 (23.12)	86 (19.77)		
Atrial fibrillation, *n* (%)			0.073	0.787
0	154 (82.8)	364 (83.68)		
1	32 (17.2)	71 (16.32)		
Hyperhomocysteinemia, *n* (%)			0.29	0.59
0	118 (63.44)	266 (61.15)		
1	68 (36.56)	169 (38.85)		
Smoking, *n* (%)			0.182	0.669
0	126 (67.74)	287 (65.98)		
1	60 (32.26)	148 (34.02)		
Alcohol consumption, *n* (%)			0.134	0.714
0	123 (66.13)	281 (64.6)		
1	63 (33.87)	154 (35.4)		
Hyperlipidemia, *n* (%)			4.176	0.041
0	148 (79.57)	312 (71.72)		
1	38 (20.43)	123 (28.28)		

**Table 3:** Construction of a multifactor logistic model using LASSO-selected variables.

Characteristic	*B*	*SE*	OR (95% CI)	*Z*	*p*
Platelet-to-lymphocyte ratio	0.01	0.002	1.01 (1.01–1.01)	5.237	< 0.001
Average platelet volume	0.751	0.121	2.12 (1.67–2.69)	6.22	< 0.001
NIHSS	0.227	0.04	1.25 (1.16–1.36)	5.699	< 0.001

### 3.3 Model verification

**Figure 4:**
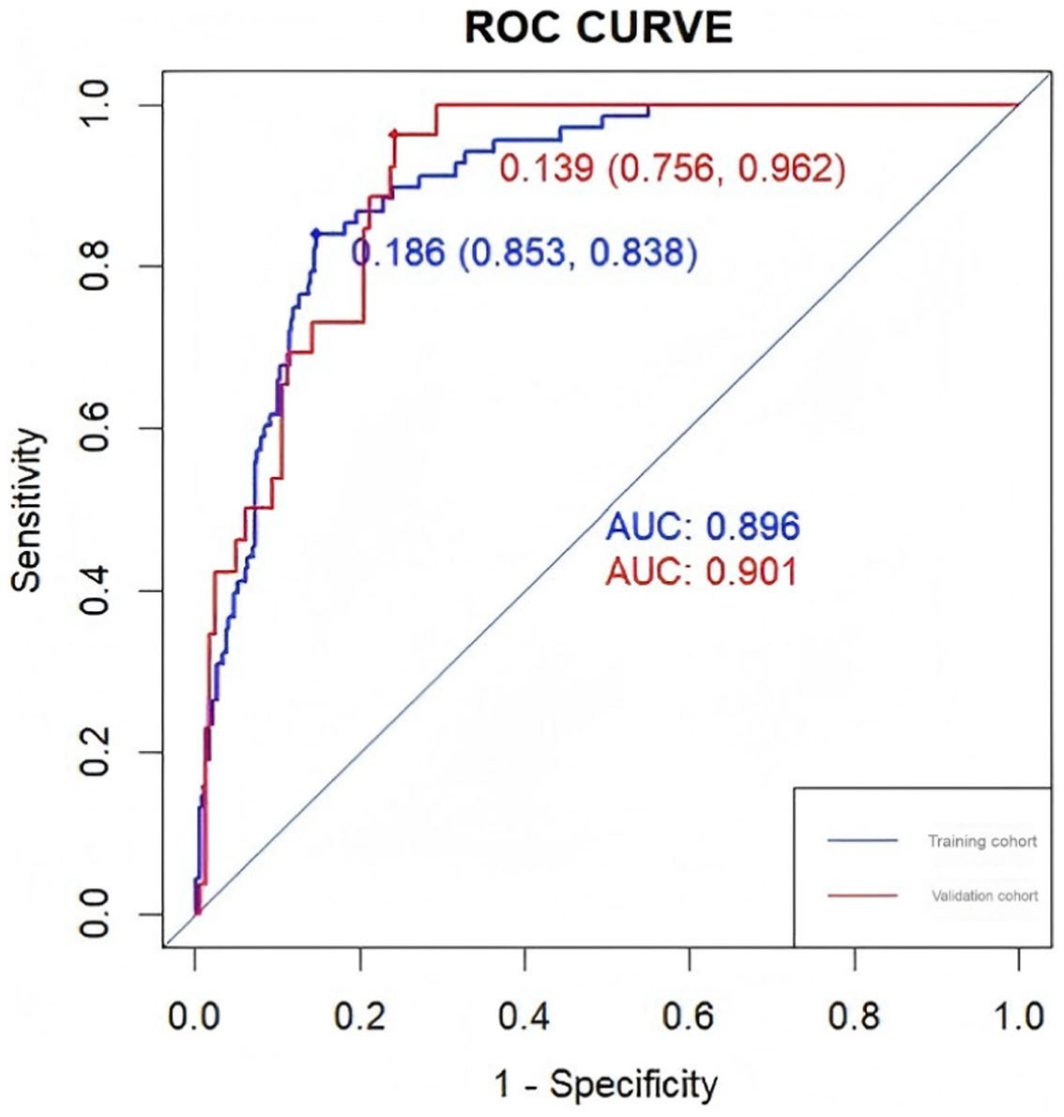
The ROC curves of the nomogram in the training and validation sets and the area under the curve are shown separately.

**Figure 5:**
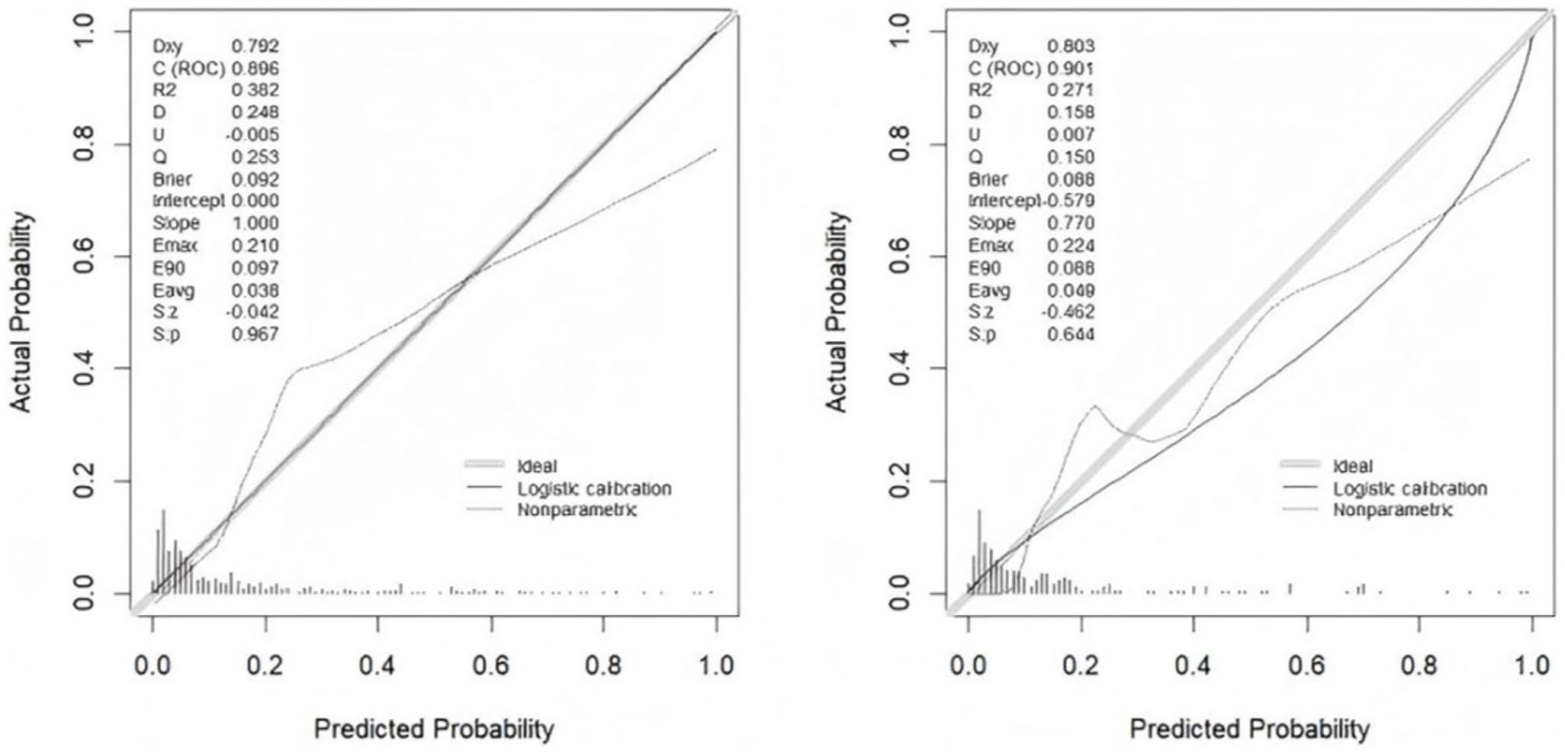
Plots of the calibration curves of the nomogram in the training set (left) and validation set (right).

**Figure 6:**
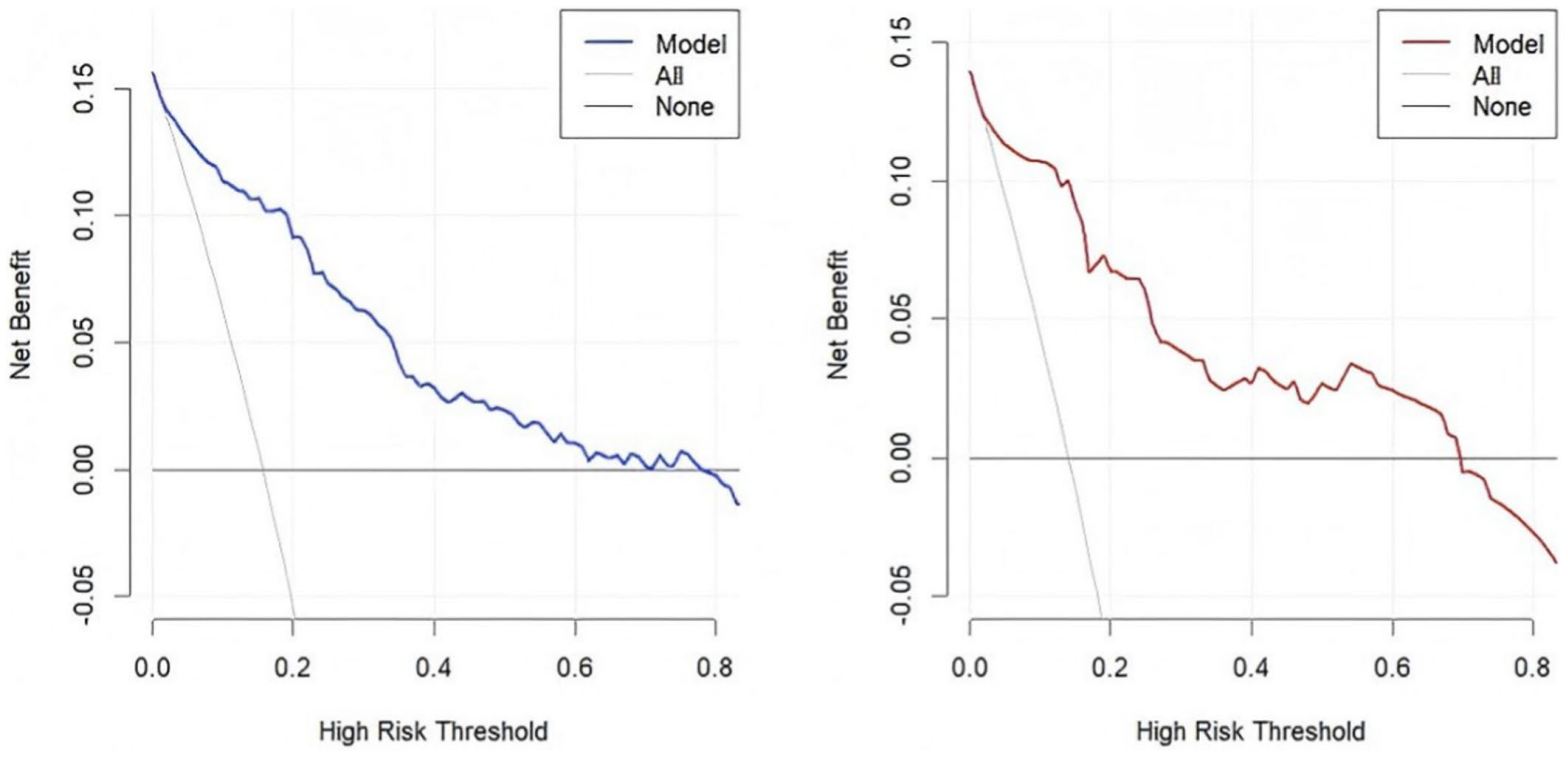
Clinical decision curves for the training (left panel) and validation sets (right panel).

The accuracy of the nomogram in predicting END was determined with ROC curve analysis (Figure 4). The results showed that the AUC (95% CI) in the training cohort was 0.896 (0.862 to 0.93), the cutoff value was 0.186, the sensitivity (SEN) was 0.838, and the specificity (SPN) was 0.858; in the validation cohort, the AUC (95% CI) was 0.901 (0.855 to 0.948), the cutoff value was 0.139, the SEN was 0.962 and the SPN was 0.756 (Table 4), indicating a satisfactory ability to differentiate early neurological functional status. Additionally, the proposed model was well calibrated (Figure 5); the Hosmer–Lemeshow test yielded a *p*-value of 0.1321 for the training set and 0.3786 for the validation set, which indicates that the predicted values from the nomogram were in good agreement with the actual values. DCA based on the training and validation cohorts showed that use of the nomogram to predict END in patients with AIS after IVT was more clinically beneficial than assuming that all patients had END or that no patients had END (Figure 6).

**Table 4:** ROC curve parameters for the training and validation sets.

Set	Cutoff	AUC	SEN	SPN
Training cohort	0.186	0.896	0.838	0.853
Validation cohort	0.139	0.901	0.962	0.756

AUC: area under the curve; SEN: sensitivity; SPN: specificity.

Cutoff denotes the critical value.

## 4 Discussion

In this study, we developed a column-line graphical model for predicting early neurological deterioration (END) in patients receiving intravenous thrombolytic therapy. Using LASSO-Cox regression methods, we screened three key variables that were easy to assess: the platelet-to-lymphocyte ratio, the mean platelet volume, and the patient’s NIHSS score at admission. With these three variables, clinicians can more accurately predict a patient’s risk of early neurological deterioration after thrombolytic therapy. The predictive model performed well in assessing the risk of END in patients undergoing intravenous thrombolysis. Through internal validation, the model showed good discriminatory and calibration abilities, exhibiting high predictive accuracy across patients. In addition, the clinical decision curve further revealed that the model has practical clinical significance at multiple probability thresholds, which implies that the model is not only theoretically valid but also capable of assisting clinicians in making more informed decisions and improving patient outcomes in practical applications. Overall, the column-line diagram developed in this study provides clinicians with a powerful tool to help identify the risk of early neurological deterioration earlier in patients undergoing intravenous thrombolysis, leading to more personalized and effective therapeutic interventions. This model could, to some extent, improve patient outcomes, improve the prognosis and reduce the incidence of adverse events.

The process of platelet activation leads to the adhesion of blood cells to the endothelial cell layer, where they aggregate to form thrombi and release many spasmogenic substances. The activation of these substances and platelets causes a dispersed blockage within the microvessels, increases the permeability of the vessels and leads to the formation of edema. This process is closely associated with the segmental occlusion of atherosclerotic cerebral arteries and vasospasm of the microvasculature (6), a phenomenon that has been extensively documented in relevant studies. Yamazaki et al. (7) noted significant differences in the expression of adhesion molecules on platelet membranes in patients with different subtypes of ischemic stroke, revealing the complex roles of these molecules in the progression of the disease. An analysis of blood levels of platelet-leukocyte aggregates by flow cytometry allowed the identification of platelet-monocyte aggregates as a sensitive indicator of platelet activation in patients with cerebral infarction (8). A study by Thornton et al. (9) demonstrated through animal experiments that IL-1 alpha plays a key role in platelet activation in cerebral endothelial cells, a step that involves leukocyte entry into brain tissue and the modulation of important processes, and the results showed that leukocytes are a major contributor to inflammation-mediated brain damage. In the early stages after focal ischemia, especially in the absence of reperfusion, many inflammatory cells infiltrate the brain and cause further damage to the brain tissue. In the context of cerebrovascular diseases (10), lymphocytes, important components of the peripheral immune system, are involved in a variety of pathophysiological processes secondary to brain injury, including neuroinflammation, neuronal necrosis, and cerebral edema (11). A study by Kim et al. (12) revealed that elevated serum levels of IL-4, IL-6, and IgE in humans may be important markers of the acute phase of cerebral infarction. However, a study by Kim et al. (13) revealed that lower lymphocyte counts are significantly associated with less of an improvement in patients’ condition in the first week after admission and a poorer functional prognosis after three months. In addition, a study by Gong et al. (14) revealed that the PLR was an independent predictor of early neurological deterioration (END), which was also strongly associated with the development of END after intravenous thrombolytic therapy. These findings further support the importance of the PLR in the diagnosis and prognostic assessment of cerebrovascular disease, providing new perspectives and approaches to understand and respond to this complex disease process.

The MPV is thought to play an important role in a variety of cardiovascular diseases, including hypertension, peripheral arterial disease, and stroke. These diseases usually involve inflammation and thrombosis, and the MPV is a key factor connecting the two (15). According to Bath et al. (16), the MPV was found to be positively correlated with the risk of stroke. Specifically, when the MPV increased by 11%, the corresponding risk of stroke increased significantly. Thus, the MPV is considered an independent predictor of stroke risk in patients with a history of stroke or transient ischemic attack (TIA). Further studies have shown that an increase in the baseline MPV is strongly associated with an increased risk of sustained epileptic disorders (END). Especially for patients with acute mild cerebral infarction treated with IVT, an elevated MPV is not only an independent risk factor for END but also strongly associated with poor prognosis at 3 months (17). These findings generally support the results of the present study, suggesting that the MPV can serve as a valid indicator for assessing the risk of stroke and its related complications. This also suggests that clinicians should fully consider the MPV as an important measure in the diagnosis and treatment of related diseases for more accurate risk assessment and early intervention.

In the present study, the NIHSS score at admission was an independent factor influencing early neurological deterioration (END) after intravenous thrombolytic therapy (IVT). In clinical practice, the NIHSS score is commonly used to assess the degree of neurological deficits in patients with acute ischemic stroke (AIS). Patients with higher baseline NIHSS scores usually exhibit more severe neurological deficits and are more critically ill. In these patients, END is more likely to occur due to the increased difficulty of revascularization with longer thrombolysis times and a relatively higher risk of bleeding. Studies have shown that the higher the NIHSS score at admission is, the greater the risk of early neurological deterioration in patients (18, 19), which is one of the main clinical features affecting END. Specifically, some studies suggest that the NIHSS score at admission is positively associated with the risk of early neurological deterioration in patients, i.e., the higher the score is, the higher the risk. Interestingly, however, a study by Sykora et al. (20) revealed that intravenous thrombolysis did not significantly increase the likelihood of a favorable prognosis in patients with an NIHSS score of 0–1 at admission. This finding implies that the benefit of IV thrombolysis may be limited in patients with milder AIS and that more caution is needed in the decision-making process to avoid unnecessary risks. In conclusion, this study highlights the importance of the NIHSS score at admission in predicting early neurological deterioration after IVT and suggests that patients’ baseline NIHSS scores and associated risk assessments should be fully considered when deciding whether to undergo IV thrombolytic therapy.

In summary, this study showed that a prediction model constructed based on the LASSO-Cox regression algorithm was highly effective at predicting the risk of early neurological deterioration (END) after intravenous thrombolytic therapy in patients with acute ischemic stroke (AIS). The application of this model can not only improve the accuracy of predicting the risk of END in AIS patients but also provide a scientific basis for early prevention and intervention in clinical practice, which can improve the prognosis of patients. Notably, the occurrence of END in patients with AIS was closely related to numerous factors, the most important of which included the platelet-to-lymphocyte ratio, mean platelet volume, and National Institutes of Health Stroke Scale (NIHSS) score at admission. These factors are highly valuable in clinical practice, and medical staff should strictly monitor and manage these indicators and implement early intervention measures to prevent the deterioration of patients’ conditions and improve treatment outcomes. However, this study also has certain limitations, which need to be overcome and improved upon in future studies. First, this study is a single-center retrospective study with a relatively limited data sample size, and the generalizability and reliability of the results need to be verified in larger, multicenter, prospective studies. Second, this study included only some of the biochemical characteristics of the patients at the time of admission and thus may have missed other biochemical indicators that have important impacts on the occurrence of END, which limits the comprehensiveness and accuracy of the model. Future studies should focus on expanding the sample size and study scope to include more centers and patient data to validate and optimize the effectiveness of the prediction model. Moreover, patients’ biochemical indicators should be comprehensively collected and analyzed to ensure that the model can more accurately reflect the various factors that influence the occurrence of END in AIS patients. Through these efforts, the treatment effect and prognosis of patients with acute ischemic stroke are expected to be further improved.

## Data Availability

The raw data supporting the conclusions of this article will be made available by the authors, without undue reservation.
